# Selective impact of CDK4/6 suppression on patient-derived models of pancreatic cancer

**DOI:** 10.18632/oncotarget.3819

**Published:** 2015-04-14

**Authors:** Agnieszka K. Witkiewicz, Nicholas A. Borja, Jorge Franco, Jonathan R. Brody, Charles J. Yeo, John Mansour, Michael A. Choti, Peter McCue, Erik S. Knudsen

**Affiliations:** ^1^ Department of Pathology, UT Southwestern Medical Center, Dallas, TX, USA; ^2^ Simmons Cancer Center, UT Southwestern Medical Center, Dallas, TX, USA; ^3^ Department of Surgery, UT Southwestern Medical Center, Dallas, TX, USA; ^4^ Department of Pathology, Thomas Jefferson University, Philadelphia, PA, USA; ^5^ Department of Surgery, Thomas Jefferson University, Philadelphia, PA, USA

**Keywords:** CDK4/6, pancreatic cancer, PD-0332991, patient-derived xenograft, tumor explant

## Abstract

Pancreatic ductal adenocarcinoma (PDA) harbors an exceedingly poor prognosis, and is generally considered a therapy-recalcitrant disease due to poor response to conventional chemotherapy coupled with non-actionable genetic drivers (e.g. KRAS mutations). However, PDA frequently loses p16ink4a, thereby leading to deregulation of CDK4/6. Surprisingly, in established cell models and xenografts, CDK4/6 inhibition has a modest effect on proliferation and resistance develops rapidly. To determine if such weak response was an intrinsic feature of PDA, we developed primary tumor explants that maintain the tumor environment and recapitulate feuture of primary PDA. The CDK4/6 inhibitor PD-0332991 was highly efficient at suppressing proliferation in 14 of the 15 explants. In the single resistant explant, we identified the rare loss of the RB tumor suppressor as the basis for resistance. Patient-derived xenografts (PDXs) were developed in parallel, and unlike the xenografts emerging from established cell lines, the PDXs maintained the histoarchitecture of the primary tumor. These PDXs were highly sensitive to CDK4/6 inhibition, yielding a complete suppression of PDA proliferation. Together, these data indicate that primary PDA is sensitive to CDK4/6 inhibition, that specific biomarkers can delineate intrinsic resistance, and that established cell line models may not represent an adequate means for evaluating therapeutic sensitivities.

## INTRODUCTION

Pancreatic ductal adenocarcinoma (PDA) has a poor prognosis, with 5-year overall survival of approximately 6% [1-4]. PDA is often diagnosed at a late stage, at which point a curative resection is no longer possible and the approved systemic therapies have a relatively modest impact on survival. Despite recent introduction of more effective chemotherapy regimens including FOLFIRINOX and combination of gemcitabine with Nab-paclitaxel, veritably all patients with advanced disease ultimately progress [5, 6]. The only approved targeted therapy, erlotinib in combination with gemcitabine, has added approximately 4 weeks to patient's overall survival, while other targeted agents including MEK, PI3K and mTOR inhibitors, have failed to demonstrate any efficacy in clinical studies [6-8]. Thus, there is an urgent and unmet need to develop rational, targeted approaches for the treatment of pancreatic cancer.

Genetic analysis of PDA has defined multiple oncogenic events that drive the initiation and progression of disease [9, 10]. Approximately 90% of pancreatic cancers are driven by KRAS, while the loss of the TP53 and SMAD4 tumor suppressors occurs in a large fraction of tumors. Unfortunately, these genetic events remain largely non-actionable due to the lack of drugs that selectively target these oncogenic events. An additional signature genetic/epigenetic event in PDA is the loss or silencing of the tumor suppressor CDKN2A [11]. Based on genetic analysis, mutation or homozygous deletion of CDKN2A occurs in ~50% of tumors, and epigenetic silencing is also frequently observed. Thus, the vast majority of PDA cases have functional loss of this tumor suppressor [8, 12].

The CDKN2A gene encodes the p16ink4a protein, which is a potent physiological inhibitor of CDK4/6 [13]. During cell cycle progression, CDK4/6 is activated downstream of multiple signaling cascades, and is required to initiate the phosphorylation of RB and subsequent entry into S-phase [14, 15]. The levels of p16ink4a are stimulated during specific stresses, leading to a blockade of proliferation and inducing features of senescence [13]. In particular, p16ink4a serves as a critical mediator of oncogene-induced senescence driven by KRAS mutation; therefore, there is strong selective pressure for its loss in PDA [16, 17]. Consequently, it is believed that the loss of p16ink4a is a key hallmark of RAS driven tumors and is frequently observed in a host of tumors with deregulation of the KRAS/RAF oncogenic singnaling pathway including non-small cell lung cancer, melanoma and colorectal cancer, in addition to PDA [18-20].

Since p16ink4a functions as an inhibitor of CDK4/6, it would be anticipated that this cell cycle regulated kinase family would be an important and actionable target in PDA. Over the last several years, drugs specifically targeting CDK4/6 have been developed [21, 22]. Presently, three highly-specific agents are now amenable to clinical use (PD-0332991, LEE-011, and LY2835219). These agents induce cell cycle arrest and delay progression/proliferation in tumor models [23-25]. This has been observed across a host of models and disease indications, including leukemias, breast cancer, and pediatric tumor types [21]. Surprisingly, in work published by several groups, single agent CDK4/6 inhibition had a particularly weak effect in models of pancreatic cancer [26-28]. Given the genetics of the disease, we sought to use patient-derived models to better delineate the significance of CDK4/6 inhibition in the treatment of PDA.

## RESULTS

### Established models of PDA have a relatively weak response to CDK4/6 inhibition

**Figure 1:**
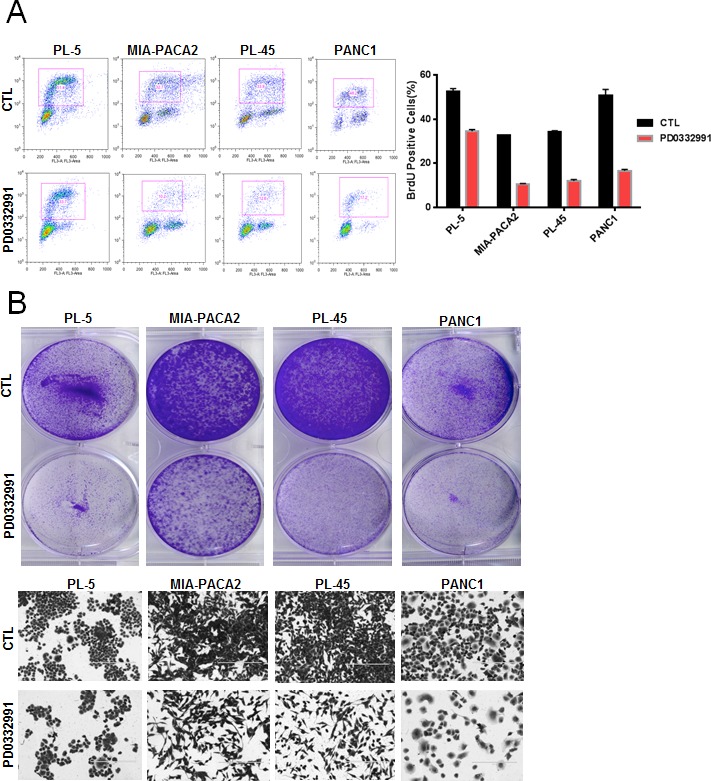

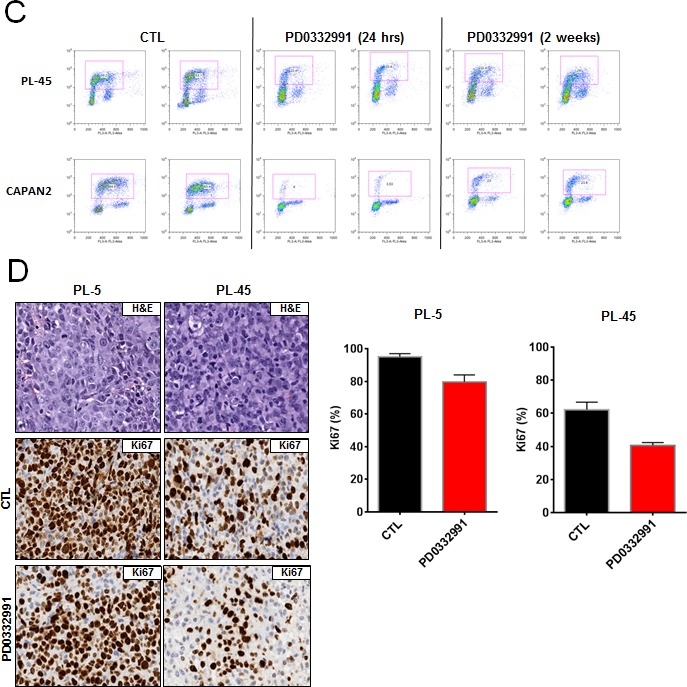
CDK4/6 inhibition yields modest efficacy in established models of PDA **A**. Representative plots of BrdU incorporation in established PDA cell lines treated with PD-0332991 (1 μM) versus control (left panel). Quantification by flow cytometry of BrdU incorporation between control and PD-0332991 treated established cell lines (right panel). **B**. Crystal violet staining of established PDA cell lines after 7 days of treatment with control vs. PD-0332991 (upper panel). Photomicrographs of PDA cells in culture after 7 days of treatment with control vs. PD-0332991 (bottom panel). **C**. Representative BrdU incorporation after 24 hours and after 2 weeks of treatment with PD-0332991. **D**. H&E and Ki-67 staining of established PDA cell line xenografts treated with control and PD-0332991. Quantification of Ki-67 staining in established PDA cell line xenografts treated with PD-0332991 and control.

In order to evaluate the response to CDK4/6 inhibition, several well-established PDA cell lines were employed. Treatment with 1 μM PD-0332991 resulted in a modest suppression of proliferation, as measured by 5-bromo-2deoxyuridine (BrdU) incorporation, in established pancreatic cancer cell lines at 24 hours of treatment (Figure 1A). In these same cell lines, crystal violet staining after 7 days of treatment demonstrated variable inhibition of tumor cell growth amongst the PDA established lines, with the PANC1 line appearing relatively sensitive to CDK4/6 inhibition and the MIA-PACA2 line appearing relatively resistant (Figure 1B). Interestingly, BrdU incorporation in cell lines treated with PD-0332991 over a span of 2 weeks demonstrated proliferation in the presence of the agent, suggesting a durable response is not achieved in these models and there is rapid progression to a resistance (Figure 1C). To determine if this phenotype was selective to cells in culture, PL-45 and PL-5 xenografts were developed. The mice were treated with PD-0332991 via oral gavage. The treatment with PD-0332991 did elicit suppression of tumor growth in PL-5 xenografts, with tumor volume in the treatment group significantly lower than that of controls (p < 0.0001). However, the suppression of residual tumor proliferation was relatively limited and even in the presence of daily dosing, a proliferative index of 79.6% in PL-5 xenografts and 40.6% in PL-45 xenografts was observed (Figure 1D). Together, these data in established models is consistent with recently published studies that suggests that monotherapy with CDK4/6 inhibitors may not be particularly effective in PDA [26-28].

### Explants cultures recapitulate the diversity of PDA

**Figure 2:**
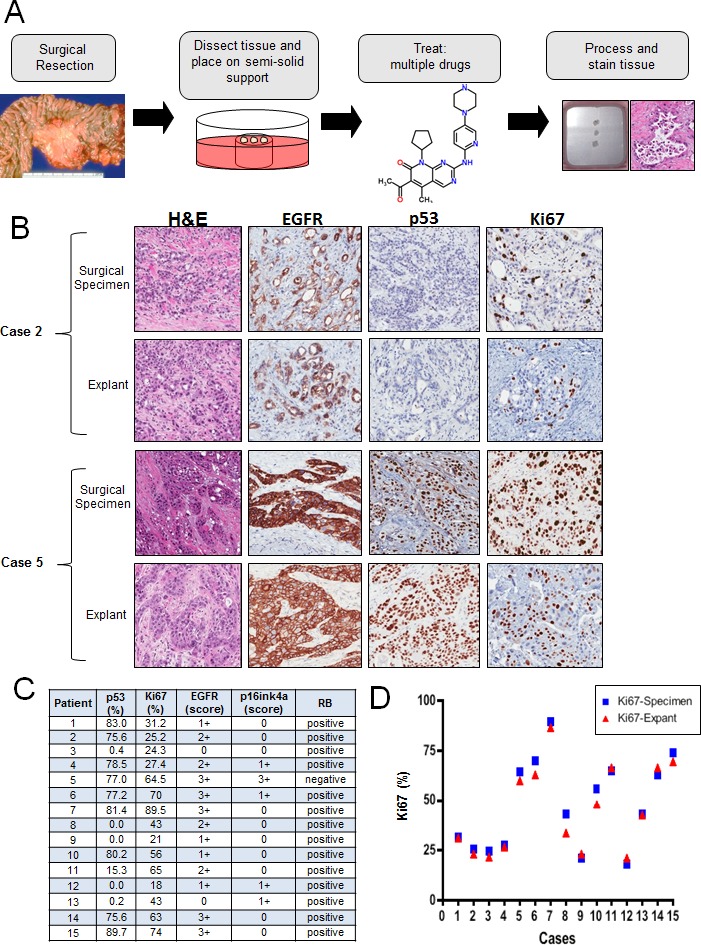
Primary tumor explants recapitulate multiple biological features of the disease **A**. Schematic representation of the explant approach. **B**. Representative hematoxylin/eosin, EGFR, p53 and Ki67 staining between surgical specimens and tumor explants. **C**. Table summarizing the status of EGFR, p16, p53, RB and Ki67 index for the cases analyzed. **D.** Quantification of Ki67 staining from matched explant and surgical specimens demonstrating concordance.

Many established cell lines have experienced selection for growth in culture, which is known to selectively perturb cell cycle regulatory mechanisms [29]. Furthermore, the desmoplastic architecture of PDA contributed by neighboring stromal cells is known to be particularly important for its biology [30, 31]. Therefore, we sought to develop a primary tumor explant model as a means to accurately recapitulate key features of PDA. The protocol used here was based on prior work with breast cancer specimens [32]. Fresh surgical tissue, not required for diagnosis, was dissected into approximately ~1 mm^3^ sections. These were cultured on a semi-solid support from 0-192 hours, during which time drugs could be applied. Explant cultures where then removed, fixed, and stained for relevant markers. After the establishment of this methodology, 15 unselected, consecutive PDA specimens were studied as explants (Figure 2A). The clinical and histopathological data of the subjects are summarized in Supplemental Figure 1. All explants retained tissue architecture and degrees of cellularity comparable to that observed in the tumor section obtained for clinical pathologic evaluation (Figure 2B). Morphological and architectural integrity of tissues was maintained in tumor samples for up to 192 hours of culture (Supplemental Figure 2A). The proliferative index was assessed by Ki67 immunostaining on day 1, 4, 6 and 8. The percentage of proliferating cells was stable across all time points, without a significant decrease up to 192 h (Supplemental Figure 2A). The number of apoptotic cells, visualized using cleaved caspase 3 staining also did not change significantly over time (Supplemental Figure 2A). The level of key molecular markers (p16ink4a, p53, EGFR) in the pathogenesis of PDA were evaluated in both primary tumor and explant, with representative staining between the surgical specimen and the cultured explants as shown (Figure 2B). P16ink4a loss or weak expression was seen in 14 of 15 cases (93%); strong p16 expression was seen in only one case (Case #5; Figure 2B). The *TP53* tumor suppressor gene is frequently inactivated in PDA, with approximately 50% of these carcinomas showing intragenic mutation. Strong nuclear labeling for the p53 protein, which correlates with mutation stabilizing p53 protein, was present in 67% of primary tumors and corresponding explants cultures (Figure 2C). Strong membranous EGFR expression (score 3+), which stimulates downstream MAPK and PI3K/AKT signaling cascades to influence cell proliferation and metastasis [33], was seen in 5 of 15 (33.3%) of primary tumors and explants cultures. These findings demonstrate that *ex vivo* explants maintain the overall biomarker phenotype of the tumor. Given the theoretical possibility of high intra-explant variability, we evaluated six explant cases from duplicate and triplicate culture wells at 48 hours. As shown in Supplemental Figure 2B, there was significant concordance between multiple explants from a given case. To determine if the explant cultures retained the same proliferative nature of the primary tumor, Ki67 was evaluated in parallel. Among primary tumors, a range from 21 to 89.5% of Ki67 positive tumor cells was observed (Figure 2C). There was a strong positive correlation in Ki67 index between the original tumor and the corresponding explanted tissue in all cases (R^2^ = 0.97, p < 0.001). These data indicate that the *ex vivo* explants retained the proliferative potential similar to that of the primary tumor.

### CDK4/6 inhibition has potent activity in patient explants

**Figure 3:**
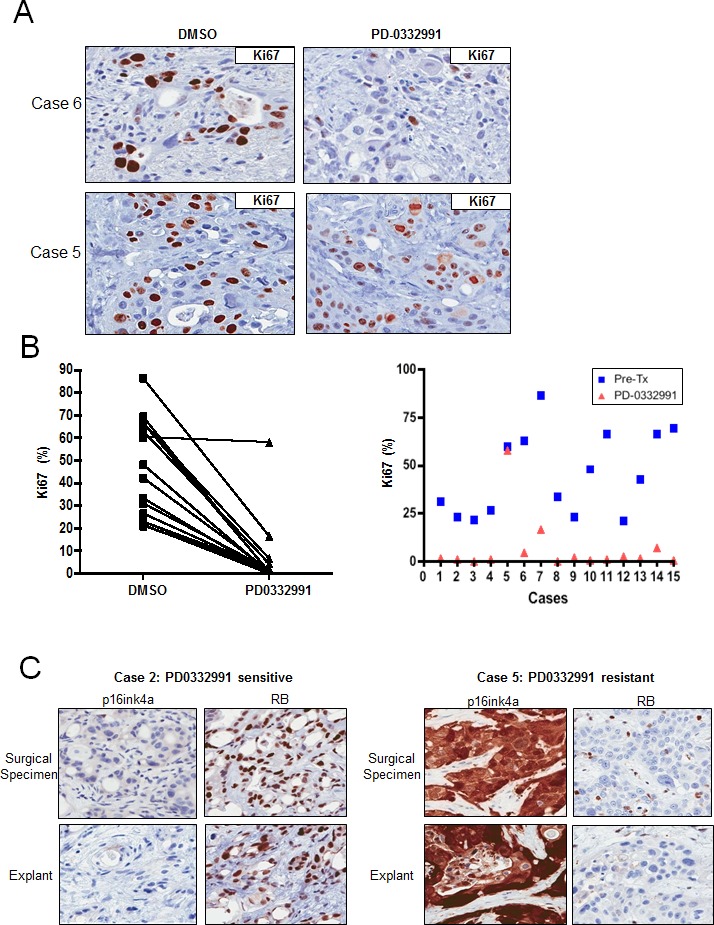
Tumor explants exhibit sensitivity to PD-0332991 **A**. Representative staining of Ki67 in drug-treated explants that exhibited significant response or lack of response (bottom panel) to PD-0332991 treatment (1 uM). **B.** Response to PD-0332991 across all explant cases evaluated. **C.** Representative staining for RB and p16ink4a in cases that responded (left panel) or did not respond (right panel) to PD-0332991.

Preservation of tissue architecture, viability and molecular phenotype suggested that treatment of explant cultures with therapeutic agents could reveal sensitivities that would be concordant with the primary tumor. In PDA explants, treatment with PD-0332991 showed profound suppression of Ki67 staining (Figure 3A and 3B). This effect was specific to the drug and was not observed in tissues treated with DMSO (vehicle) for the same period or with gemcitabine (Supplemental Figure 3A). Interestingly, there was some heterogeneity in the response to PD-0332991. Specifically, 13 of the 15 cases exhibited a greater than 5-fold suppression of Ki67 upon exposure to PD-0332991, and post-treatment Ki67 index below 7% (Figure 3B). In contrast, one case maintained a Ki67 index of 16.5% post-treatment, in spite of a greater than 5-fold reduction (case #7), and one case showed no reduction in Ki67 staining (case #5). Of note, the response to PD-0332991 did not depend on p53 status but was specific to the tumor cells, since lymphocytes present within the explants effectively responded as evidenced by suppression of Ki67 (not shown). Thus, the failure to respond to PD-0332991 appears to be a consequence of tumor-specific genetic events, and not due to distinctions in intrinsic drug sensitivity of a given individual. Since preclinical data suggest that an intact RB pathway is required for the cytostatic response to PD-0332991 [34-37], we further investigated the activation status of the RB pathway by immunohistochemical stains. Tumors that are RB deficient expressed exceedingly high levels of p16ink4a with high Ki67 proliferation index [13, 32]. Both the primary tumors and explants were stained for p16ink4a and RB. Within the cohort of evaluated cases, only one case exhibited high levels of p16ink4a and low RB (Case #5; Figure 3C). RB and p16ink4a expression was conserved between the primary tumors and the explants. These data indicate that the vast majority of PDA is responsive to PD-0332991 and only the infrequent loss of RB is associated with lack of response.

### Suppression of tumor growth in patient derived xenografts

**Figure 4:**
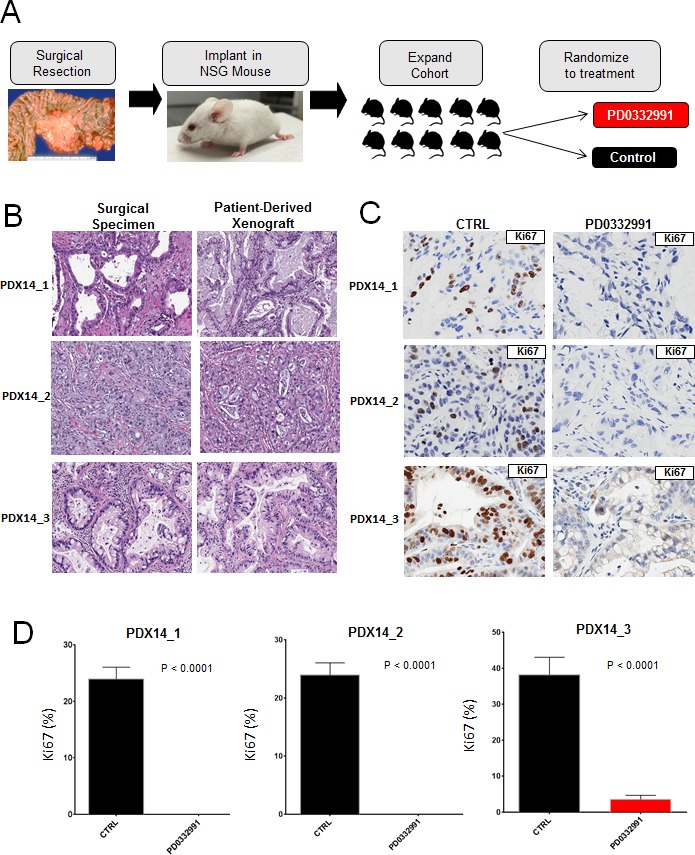

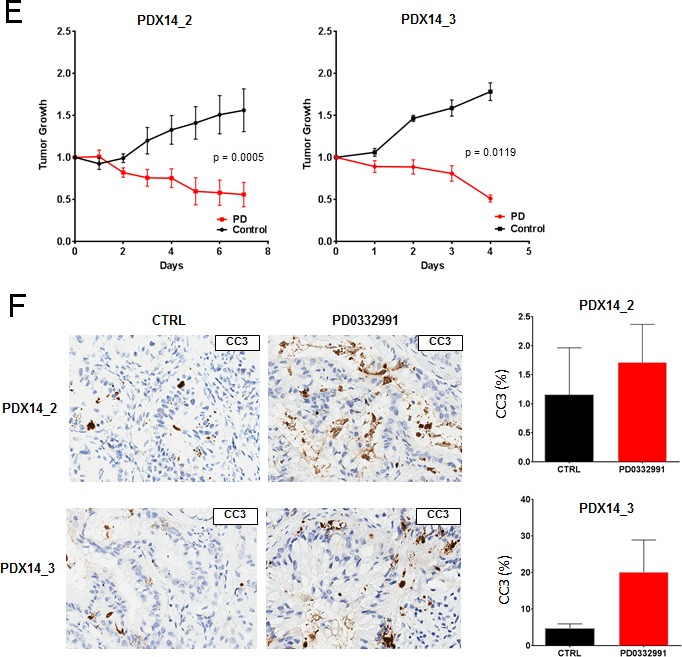
Patient-derived xenografts recapitulate primary tumor and demonstrate significant vulnerability to CDK4/6 inhibition **A**. Schematic representation of the patient-derived xenograft approach. **B.** Representative hematoxylin/eosin staining between surgical specimens and patient-derived xenografts. **C**. Representative staining of Ki67 in control and PD-0332991 treated PDXs. **D**. Quantitation of Ki67 in the indicated PDX. **E.** Tumor growth of PDXs, normalized to initial tumor volume, while treated with PD-0332991 or control. **F**. Representative staining of cleaved caspase 3 in both control and PD-0332991 treated PDXs.

Though tumor explants provide a striking recapitulation of the primary tumor, we sought to complement this approach with patient-derived xenografts to obtain *in vivo* data over a longer treatment period. In this model, fragments of primary tumor are subcutaneously implanted in NSG mice, allowing the tumors to grow and subsequently passaged into other NSG mice for expansion into treatment cohorts (Figure 4A). Unlike the PL-45 or PL-5 xenografts that harbor little tissue architecture reminiscent of PDA, the three patient-derived xenograft exhibited histologic architecture remarkably similar to their tumor of origin (Figure 4B). Treatment of the cohort with PD-0332991 resulted in marked suppression of tumor growth and in some cases a reduction in tumor volume (Figure 4C). Analysis of the tissue treated with PD-0332991 revealed complete suppression of Ki67 across all three PDX cohorts (Figure 4D). This profound anti-proliferative effect on Ki67 was not seen in the PDX cohort treated with gemcitabine similar to the observations in explant culture (Supplemental Figure 3B). Each tumor had a low baseline of apoptosis, as observed with cleaved caspase 3, and PD-0332991 either had no effect or elicited a slight increase in tumor cell turnover (Figure 4E). Together, these data indicate that CDK4/6 suppression is particularly effective in limiting the proliferation of patient-derived models of resectable disease.

## DISCUSSION

Treatment of PDA continues to present a significant challenge and in spite of multiple preclinical studies there has been only modest improvement in survival, with the principle gains coming from cytotoxic chemotherapies [1]. The vast majority of preclinical studies supporting drug development are carried out in established cell line models grown in culture or as xenografts. While facilitating rapid analysis, these models are not entirely representative of PDA. Many of these models, as shown here, grow very rapidly in cell culture and correspondingly produce xenografts with higher Ki67 indices than those reported in clinical PDA samples. The average Ki67 index in primary PDA is ~30% and the tumor exhibits an organized glandular architecture [38]. Additionally, the tumor is dominated by desmoplastic stroma that has recently emerged as a tumor suppressive feature of the disease though it also inhibits drug uptake [30, 39-41]. Despite the established models harboring genetic alterations that would be expected to confer substantial sensitivity to targeting CDK4/6, xenografts derived from established cell lines rapidly progress. This may reflect decades of selection for cell cycle deregulation in culture conditions. As result, high proliferation index and possibly cell cycle deregulation in these models can simply overwhelm cytostatic/targeted therapies through mechanisms that may not reflect primary tumor biology. While our study focused on CDK4/6 inhibition, cell cycle responses influence the activity of a range of targeted agents [42, 43], and this observation could be relevant to many targeted drug therapies.

It has become well accepted that tumors regulate the development of surrounding stroma through the induction of growth factor receptors and the production of their ligands [41, 44, 45], and that there is a degree of reciprocity involved in this communication, i.e., tumor stroma can influence tumor epithelial cells. The mutual interaction between tumor cells and surrounding environment appears particularly significant in PDA, where stromal cell promote tumor development and invasiveness but, in some cases, also limit rampant tumor cell growth [30, 46-48]. Thus, it is of importance to develop models that recapitulate these complex interactions. We employed two models that allowed us to study therapeutic efficacy from patient specimens: *ex vivo* tissue explants cultures and patient-derived xenografts. The histological features of both models are highly distinct from the fairly uniform collection of cells seen in xenografts established through injections of tumor lines such as PL-45 and PL-5. However, each of these models has intrinsic advantages/disadvantages. The explant model provides a window into the intact tumor of the patient. It fully retains human tumor-stroma interactions, which allows for studying the activity of anticancer drugs and small molecule inhibitors in the context of an individual patient's tumor biology-in some ways reproducing the effect of performing serial biopsies to assess tissue response to therapy. In spite of the desirable features of the explant model, it does not recapitulate mechanisms of drug delivery, metabolism, and *in vivo* physiology. The PDX models employed accurately recapitulate the histological features of the pancreatic cancer from which they were derived and allow the study of tumor growth and inhibition over time. In the era of personalized medicine, these models could allow genetic alterations in tumor cells to be targeted within the context of their complex interactions with the stromal compartment.

Targeting cell cycle control is an attractive anti-tumor strategy, with the goal of preventing disease progression. The vast majority of human tumors exhibit some form of cell cycle deregulation, allowing tumors to proliferate unabated. Multiple aberrations affect the G1/S transition, including the amplification of D1 cyclin, deletions and mutations of RB and/or downregulation of CDK4/6 inhibitor p16ink4a [49, 50]. All these events lead to loss of proliferative control, with p16ink4a inactivation being exceedingly common in PDA [3, 13, 51]. Thus, it was unexpected that multiple established pancreatic cancer cell models would exhibit a relatively weak response to pharmacological CDK4/6 inhibit. In contrast, all explants that retained RB protein and showed low p16ink4A staining exhibited decrease in Ki67 proliferation index upon treatment with the CDK4/6 inhibitor PD-0332991. While RB loss has not been previously reported in pancreatic cancer cell models, the data here demonstrates that in some PDA cases functional expression of RB is lost. These data also indicate that the analyses of p16ink4a and RB status can be used prospectively to evaluate the response to CDK4/6 inhibitors in clinical specimens, and suggests an approach for developing markers for targeted therapies prior to clinical trials. Moreover, cytostatic response to PD-0332991 was confirmed in PDX models, lending further support to the possibility that CDK4/6 inhibitors may have potent activity in PDA.

A key question is whether the distinct response to PD-0332991 between established and patient-derived models reflects intrinsic PDA biology. While established models were initially developed from PDA lesions, some originated from metastatic disease, and most have been serially cultured for long periods of time, wherein mutations that enhance proliferation are progressively selected. It remains unclear if changes acquired during passaging *in vitro* mirror mutations occurring in primary tumor post chemotherapy and/or during progression to metastatic disease. The work herein demonstrates that our patient-derived models of PDA share remarkably similar biology to their pancreatic tumor of origin, and that in these models, CDK4/6 inhibition leads to potent suppression of tumor growth. Despite limited efficacy in established cell line models, this data suggests that CDK4/6 inhibitors should be considered for inclusion into emerging pancreatic cancer clinical trials.

## MATERIALS AND METHODS

### Cell culture, derivation and treatment

The established cell lines PL-45, MIA-PACA2, PL-5, PANC1, and CAPAN2 were cultured in DMEM supplemented with 10% FBS, as previously published [28]. Cells were treated with 1 μM CDK4/6 inhibitor PD-0332991, with drug replenished every 72 hours. Acute and durable responses to PD-0332991, measured at 24 hours and 2 weeks respectively, were assessed by 5-bromo-2-deoxyuridine (BrdU) incorporation and measured by flow cytometry. Following conclusion of treatment, PDA cell models were fixed in ice-cold 70% ethanol (in PBS) for 2 hours at 4^º^C. Fixed cells were incubated with 2 M HCl and 0.3 mg/ml pepsin for 30 minutes at room temperature and neutralized with 0.1 M sodium borate (pH 8.5) for 2 minutes. Following neutralization, cells were washed with IFA buffer (10 mM HEPES [pH 7.4], 25 mM NaCl, 4% fetal calf serum) and then incubated in IFA containing 0.5% Tween for 10 min at room temperature. Cells were pelleted and re-suspended in FITC-anti-BrdU diluted in IFA buffer (1:10) (BD Biosciences, San Jose, CA) for 1 hour at room temperature. After antibody incubation, cells were washed once in IFA and incubated for 10 min on ice in 0.5 ml of PBS containing 20 μg/ml PI and 40 μg/ml RNase. Cells were then analyzed by FACSCalibur (BD Biosciences) instrument to determine PI (red) and FITC (green) staining for nuclear DNA and BrdU content, respectively.

### Tissue preparation and explant cultures

PDA tumor tissue from resected surgical specimens, which was not needed for diagnostic purposes, was used. The collection of tissue was approved by the institutional review board (IRB). Tissues collected for explants culture were placed into 10 mL of complete explant culture medium for transport to the laboratory. Explant culture media consisted of RPMI 1640 base (Sigma) supplemented with 10% fetal calf serum (FCS). Antibiotic/antimycotic solution was added to a final concentration of 1 X (Sigma). Hydrocortisone (Sigma) was re-suspended in 95% EtOH and supplemented at a concentration of 1mg/100ml media. Lastly, recombinant human insulin was added at a final concentration of 1mg/100ml media (Gibco). Tissues were maintained in this medium at 4^º^C until processing. All source tissues were plated as explants within 12 hours. Under sterile conditions and one hour prior to tissue dissection, 1 cm^3^ hemostatic gelatin dental sponges (Vetspon, Novartis) were hydrated in explant culture media at 37^º^C. While the sponges were hydrating, tissues were transferred to a 10 cm cell culture dish with 10 ml complete explant media for dissection. Using a sterile scalpel and microdissection scissors, any apparent fat tissue was removed from the surface of the specimen and discarded. Samples were then cut into 1 mm^3^ pieces. Upon complete hydration of gelatin sponges, each was transferred to individual wells of a 12-well cell culture dish, and 2.5 ml of complete explant media was added to each well. One mm^3^ dissected specimen sections were chosen at random and placed on top of each sponge using sterile forceps. Tissues were placed near the edges of the sponge to allow for media perfusion in between tissue sections. Explant cultures were incubated at 37^º^C and 5% CO_2_.

Each treatment condition was examined with three pieces of tissue per sponge. Fifteen independent PDA cases were evaluated. Only 12 explant cultures were evaluated for response to gemcitabine due to limited tissue. Following plating, explants were allowed to recover in explant media for 24 hours. Explants were then treated for 48 hours with vehicle control (DMSO), a selective CDK4/6 inhibitor PD-0332991 (1 μM), or gemcitabine (20 nM). Explants were removed from culture, fixed and processed for immunohistochemical analyses.

### Histology and immunohistochemical stains

The original tumor tissue and the explant of the tumor tissue were fixed in 10% neutral-buffered formalin and embedded in paraffin. Four microm sections were stained with H&E for the histological evaluation. For immunohistochemical analysis four micron paraffin sections were prepared, deparaffinized in xylene, and rehydrated with graded ethanol. Antigen retrieval was performed by microwave pretreatment. The following antibodies were employed for the analysis: Ki67 (clone 30-9, dilution, Ventana Medical Systems), p53 (clone DO-7, dilution 1:100, DAKO), p16ink4a (clone E6H4, dilution 1:50, MTM Laboratories), RB (clone 1F8, dilution 1:50, Thermoscientific), EGFR (clone 5B7 prediluted, Ventana Medical Systems) and cleaved caspase 3 (clone 5A1E, dilution 1:300, Cell Signaling). All stains were performed using the BenchMark XT Slide Preparation System (Ventana Medical Systems).

### Automated image analyses and scoring

Immunohistochemically stained primary tumor and explant slides were scanned using an Aperio ScanScope®CS instrument at 20X, (Aperio Technologies, Vista, CA, USA). Stains were quantified using analysis tools developed by Aperio. Nuclear algorithms were used to evaluate Ki67, p53 and RB stain; membranous and cytoplasmic algorithms were applied for EGFR and p16ink4a quantification, respectively. The mean staining intensity and percentage of staining cells were recorded for Ki67, p53, RB and p16ink4a stains. Immunolabeling for p53 was interpreted as positive and predictive of TP53 mutations if >75% tumors cell showed strong positive staining. RB expression was scored as negative (cancer cells showed no staining while normal cells were positive), weak (mean staining intensity was less than adjacent normal cells), or strong (staining intensity was equal to adjacent normal cells). RB loss was defined as a score of either negative, or weak. For p16ink4a the staining was graded as 0 (no cells staining), 1+ (diffuse weak cytoplasmic expression or < 25% cell showing strong staining), 2+ (25% to 75% of cells with strong staining), and 3+ (> 75% cells showing strong staining). A 3+ score was considered high expression. For EGFR the score (range 0-3) was reported.

### Patient-derived xenografts

NOD-SCID IL2Rgamma^null^ (NSG) mice were maintained in a barrier facility under HEPA-filtered air with food and water available as desired. Food, water and cage bedding were sterilized prior to use. Mice were manipulated under sterile conditions during surgery. Animal experiments fulfilled National Institutes of Health and UT Southwestern requirements for humane animal care and were approved by the UT Southwestern Institutional Animal Care and Use Committee.

Tumor tissue acquisition was performed under an Institutional Review Board (IRB) protocol approved at UT Southwestern Medical Center. Fresh tumor tissue was stored in sterile specimen cups and transported in RPMI-1640 (Sigma-Aldrich, St. Louis, MO) on ice. The tumor specimens were dissected in 3 mm × 3 mm fragments and coated in Matrigel^®^ basement membrane matrix (Corning Life Sciences, Bedford, MA). NSG mice were anesthetized with isoflurane and, once sedated, their flank was shaved and cleansed with 70% alcohol and betadine. A 1 cm incision was made through the skin and subcutaneous tissue. A tumor fragment was then implanted into the flank and the incision was closed with 4-0 Vicryl suture. Mice were monitored daily for 2 consecutive days after surgery with particular attention paid to animal distress, wound dehiscence and signs of infections. Thereafter, they were examined 2-3 times per week. Tumor size was assessed by measurement with calipers once a week. Tumor volume was approximated using measurements of tumor length and width with the formula: (length × (width^2^))/2. The mice were sacrificed and tumors were harvested once they reached a diameter greater than 1.5 cm. The tissue was frozen in liquid N_2_ as well as kept fresh in RPMI for passaging into a larger cohort of mice. All three patient-derived xenografts were successfully passaged twice in order to develop a cohort large enough for treatment.

### Cell line xenografts

Approximately 3.0×10^6^ PL-45 and PL-5 cell lines were mixed with Matrigel® basement membrane matrix (Corning Life Sciences, Bedford, MA). Cells were injected subcutaneously into the flanks of NOD-SCID IL2Rgamma^null^ (NSG) mice. Tumor progression was measured by calipers twice weekly and tumor volume was approximated using the formula: (length × (width^2^))/2.

### Xenograft treatment

Treatment with PD-0332991 was performed on cohorts of at least 8 mice with tumors measuring between 150 and 500 mm^3^. The mice were randomized to treatment or control arms. The treated mice received palbociclib via oral gavage at a dose of 125 mg/kg in pH 4 lactate buffer (50 mM) daily. Gemcitabine was administered by intraperitoneal injection at a dose of 50 μg/kg in phosphate buffered saline. The control mice received lactate buffer via oral gavage once daily. Tumor sizes were assessed daily using calipers to determine tumor volume as described above. Treatment lasted for 5-8 days, after which the mice were sacrificed and the tumors were fixed in 10% formalin for histologic and immunohistological analysis.

### Statistical analysis

All statistical analyses were performed using GraphPad Prism (GraphPad Software, Inc). Student's t-test and Pearson correlation analyses were used and results with p < 0.05 were considered significant.

## SUPPLEMENTARY MATERIAL FIGURES


