# Supplementary material for: Selective impact of CDK4/6 suppression on patient-derived models of pancreatic cancer
Source: Oncotarget. 2015 Apr 14;6(18):15788–801. doi: 10.18632/oncotarget.3819 (PMC4599237; doi:10.18632/oncotarget.3819)

Selective impact of CDK4/6 suppression on patient-derived models of pancreatic cancer

Supplementary Material

Supplementary Figure 1. Clinical and histopathological data of the subjects

Patient	Surgical Procedure	Age (years)	Gender	Histologic Diagnosis	Histologic Grade	TNM stage
1	Pancreatoduodenectomy	58	M	Adenocarcinoma	3	IIB
2	Pancreatoduodenectomy	66	M	Adenocarcinoma	3	IIA
3	Pancreatoduodenectomy	75	M	Adenocarcinoma	3	IIB
4	Pancreatoduodenectomy	70	M	Adenocarcinoma	3	IIB
5	Pancreatoduodenectomy	65	M	Adenosquamous carcinoma	2	IIB
6	Pancreatoduodenectomy	78	F	Adenosquamous carcinoma	3	IIA
7	Pancreatoduodenectomy	68	M	Adenocarcinoma	2	IIB
8	Pancreatoduodenectomy	71	F	Adenocarcinoma	2	IIB
9	Pancreatoduodenectomy	69	M	Adenocarcinoma	2	IIB
10	Pancreatoduodenectomy	77	M	Adenocarcinoma	2	IIA
11	Pancreatoduodenectomy	76	M	Adenocarcinoma	2	IIB
12	Distal Pancreatectomy	59	F	Adenocarcinoma	2	IIA
13	Pancreatoduodenectomy	52	M	Adenocarcinoma with endocrine differentiation	3	IV
14	Distal Pancreatectomy	73	M	Adenosquamous carcinoma	2	IIA
15	Distal Pancreatectomy	66	F	Adenosquamous carcinoma	3	IIB
PDX14-1	Pancreatoduodenectomy	72	M	Adenocarcinoma	2	IIB
PDX14-2	Pancreatoduodenectomy	57	M	Adenocarcinoma	4	IIA
PDX14-3	Pancreatoduodenectomy	58	M	Adenocarcinoma	2	IIA

Supplementary Figure 2. Explants maintain proliferation and viability for 192 hours of *ex vivo* culture

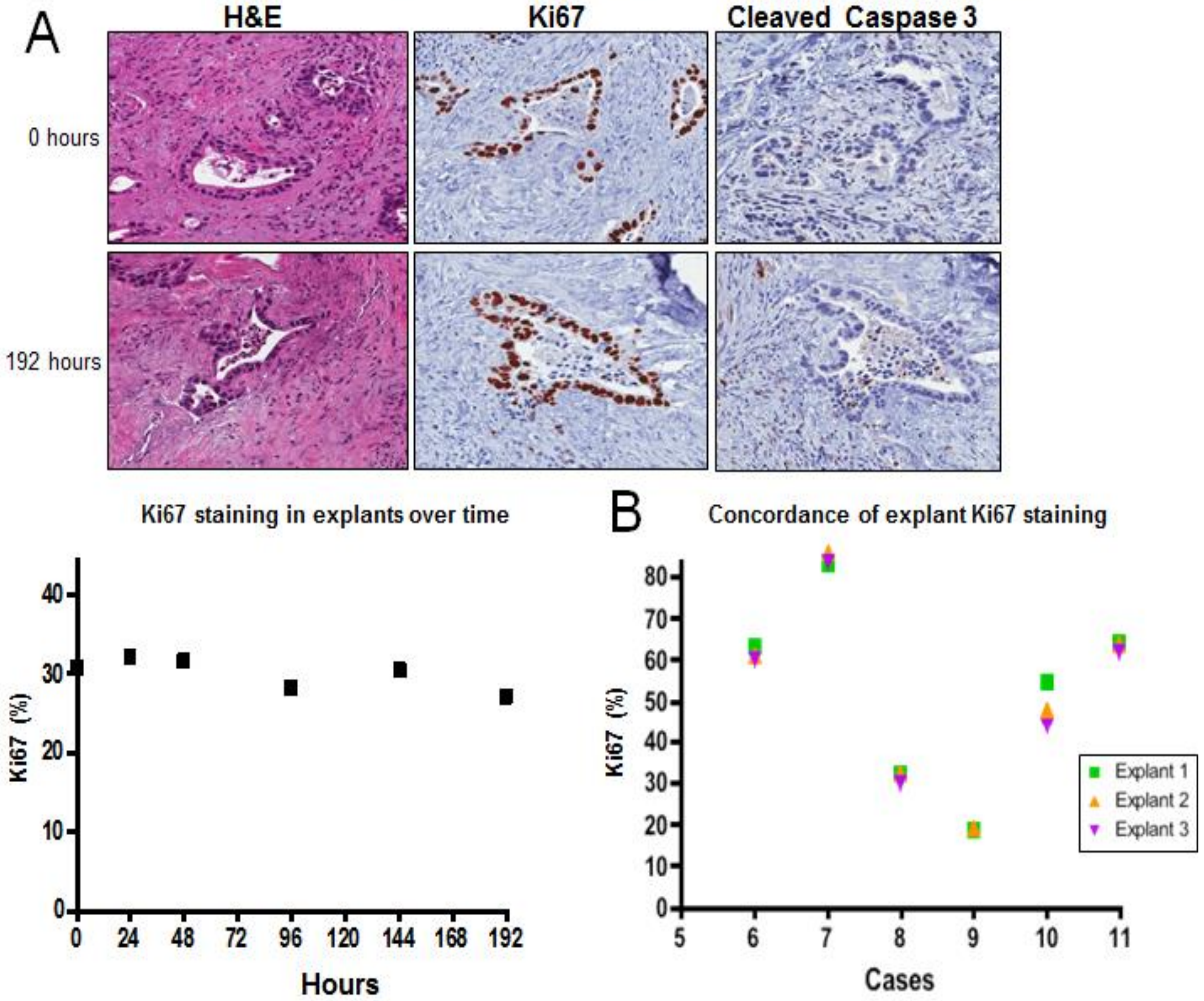

Supplementary Figure 3. Ki67 staining in explants and PDXs, post-treatment with Gemcitabine

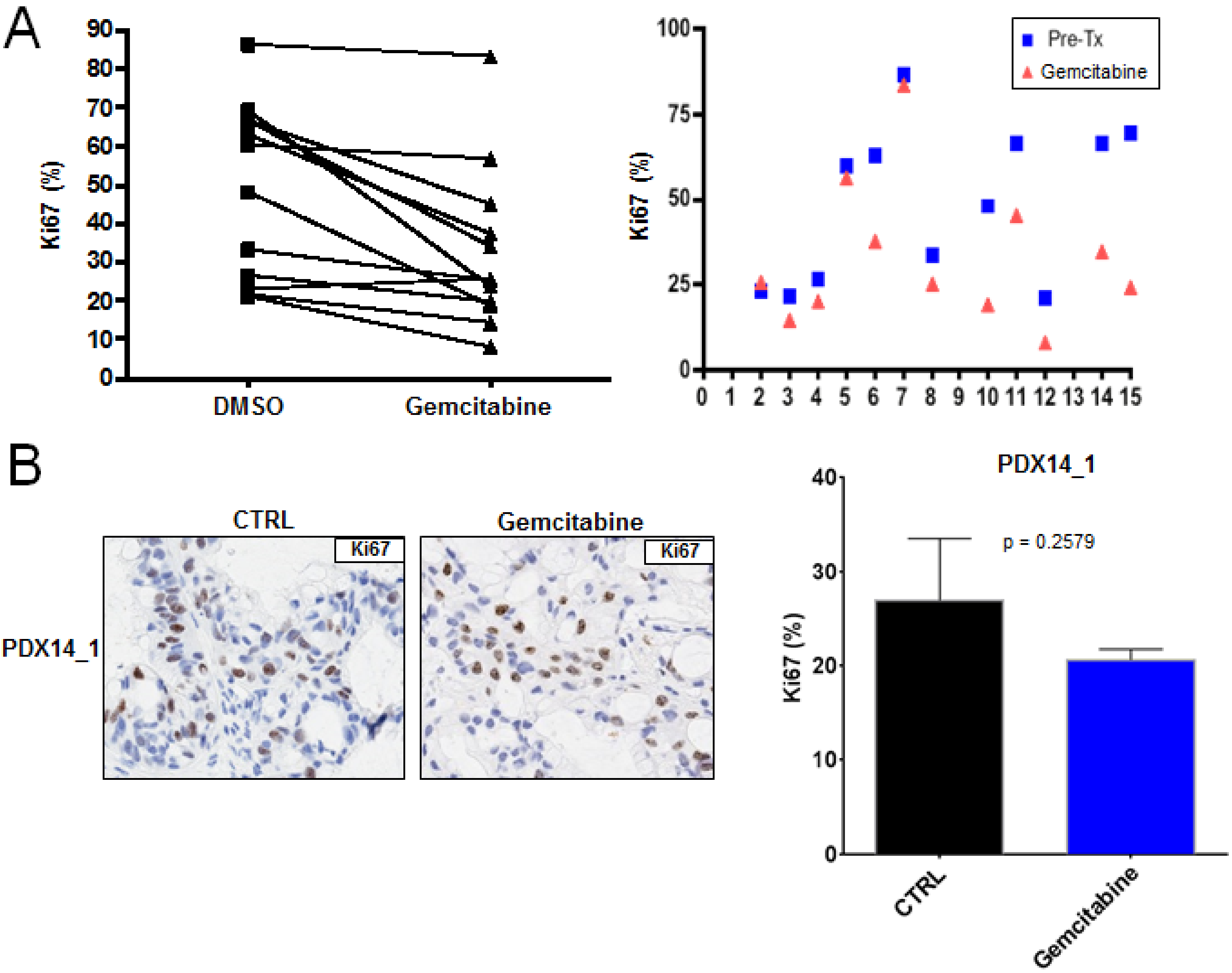

Supplement: Supplementary file 1 [file oncotarget-06-15788-s001.pdf]
